# Correlation between airborne pollen data and the risk of tick-borne encephalitis in northern Italy

**DOI:** 10.1038/s41598-023-35478-w

**Published:** 2023-05-22

**Authors:** Giovanni Marini, Valentina Tagliapietra, Fabiana Cristofolini, Antonella Cristofori, Francesca Dagostin, Maria Grazia Zuccali, Silvia Molinaro, Elena Gottardini, Annapaola Rizzoli

**Affiliations:** 1 Research and Innovation Centre, Fondazione Edmund Mach, San Michele All’Adige, TN Italy; 2 Azienda Provinciale Servizi Sanitari, Trento, Italy

**Keywords:** Ecology, Diseases

## Abstract

Tick-borne encephalitis (TBE) is caused by a flavivirus that infects animals including humans. In Europe, the TBE virus circulates enzootically in natural foci among ticks and rodent hosts. The abundance of ticks depends on the abundance of rodent hosts, which in turn depends on the availability of food resources, such as tree seeds. Trees can exhibit large inter-annual fluctuations in seed production (masting), which influences the abundance of rodents the following year, and the abundance of nymphal ticks two years later. Thus, the biology of this system predicts a 2-year time lag between masting and the incidence of tick-borne diseases such as TBE. As airborne pollen abundance is related to masting, we investigated whether inter-annual variation in pollen load could be directly correlated with inter-annual variation in the incidence of TBE in human populations with a 2-year time lag. We focused our study on the province of Trento (northern Italy), where 206 TBE cases were notified between 1992 and 2020. We tested the relationship between TBE incidence and pollen load collected from 1989 to 2020 for 7 different tree species common in our study area. Through univariate analysis we found that the pollen quantities recorded two years prior for two tree species, hop-hornbeam (*Ostrya carpinifolia*) and downy oak (*Quercus pubescens*), were positively correlated with TBE emergence (R^2^ = 0.2) while a multivariate model with both tree species better explained the variation in annual TBE incidence (R^2^ = 0.34). To the best of our knowledge, this is the first attempt at quantifying the correlation between pollen quantities and the incidence of TBE in human populations. As pollen loads are collected by widespread aerobiological networks using standardized procedures, our study could be easily replicated to test their potential as early warning system for TBE and other tick-borne diseases.

## Introduction

In Europe, zoonoses originating from wildlife reservoirs and/or transmitted by arthropods are expected to rise in the future due to environmental and climatic changes^1,2^. This is particularly true for tick-borne diseases such as Lyme borreliosis, tick-borne relapsing fever, Crimean-Congo haemorrhagic fever and tick-borne encephalitis (TBE).

TBE virus (TBEv) is a flavivirus that affects the human and animal central nervous system. This virus is mainly transmitted by the bite of an infected tick. However, TBEv can also be acquired via consumption of infected unpasteurized milk and dairy products and through other non-vectorial routes of transmission^3^. The number of human cases of TBE have been increasing from 1995 to 2020^4^ in both endemic^5^ and in previously unaffected countries, such as Belgium and the United Kingdom^6–8^. In Europe, TBE became a notifiable disease since 2012, and it is considered a major public health concern with a total of ~ 3000 human cases reported each year in 25 countries. TBEv is typically distributed in hotspots (foci of infection) characterized by high spatial and temporal variability. Numerous modelling studies have investigated how the presence of suitable habitat, climate conditions, and host availability influence the distribution of ticks and TBEv and the mechanisms of TBEv transmission^9–16^.

In its natural enzootic cycle, transmission involves ixodid ticks, mainly belonging to the genus *Ixodes*, and their small mammal hosts (rodents and insectivores). In western Europe, the castor bean tick, *Ixodes ricinus*, is the most important tick vector. The life cycle of *I. ricinus* includes four developmental stages (egg, larva, nymph, and adult), requires three blood meals, and takes an average of three years (varying from two to six). Discrete cohorts of all three ticks stages occur in the environment at the same time. Larval and nymphal ticks feed on small mammals, whereas adult ticks feed on large ungulates. In *Ixodes* ticks, TBEv has both transovarial transmission (from the mother to the eggs) and transstadial transmission (from one stage to the other). Ticks can acquire TBEv by either systemic transmission or non-systemic transmission (also known as co-feeding transmission). In systemic transmission, the infected host develops a sufficient viremia to transmit TBEv to feeding ticks. In co-feeding transmission, the host acts as a vehicle for the spatio-temporal coincidental feeding of naïve larvae and infected nymphs, and viral transmission occurs at the local skin site level^17^. Thus, co-feeding transmission requires synchronous questing of larvae and nymphs on the same vertebrate reservoir hosts. Theoretical models based on the basic reproduction number (R_0_) indicate that co-feeding transmission is most important for the persistence of TBEv in nature^18^. Hence, the synchronous activity of larvae and nymphs and the presence of rodents are crucial to maintain TBEv in nature^19^ and may explain the highly focal distribution of this pathogen compared to the wide geographic distribution of both the ticks and vertebrate hosts^9^.

Rodents are also important for feeding the immature stages of ticks (larvae and nymphs). The availability of more hosts increases the chances of larvae to feed and develop into nymphs. If the hosts are competent for TBEv then the pathogen can be amplified, thus increasing the density of infected nymphs and consequently the risk of exposure for susceptible hosts, including humans^9,14,20,21^. Increased resource availability, as a consequence of global changes, is widespread in many ecosystems strongly affecting animal communities^22^, both in terms of population dynamics and interactions between consumers and resources. Rodents are r-strategy species (short generation times, fast and high reproductive rate) that quickly react with functional and numerical responses to environmental changes such as the availability of food resources. Intra-annual seasonal fluctuations in rodent density follow the favorable vegetation period, while inter-annual outbreaks in rodent density are driven by synchronized production of seed crop termed mast^23–28^. Mast seeding is a well-known example of pulsed resources in terrestrial ecosystems. The density of granivorous rodents can increase dramatically the year after a mast event and crash two years later. Inter-annual variation in rodent density and their pathogens will influence the zoonotic risk to humans but the timing of increased risk relative to the mast event will differ among pathogens^29^. Thus, when the pathogen is transmitted by a tick with a multi-year life cycle, high rodent density caused by larger food availability leads to an increase in the number of infected nymphs the following year, and hence a greater incidence of human cases is expected to occur two years after the masting event^5,12,13,30–32^.

The link between tree masting, rodent population dynamics, density of nymphal ticks and eventually the incidence of tick-borne diseases in humans, has been investigated in several studies mainly correlating some climatic variables that regulate the mast event^33,34^ or directly using a masting index to predict the incidence of TBE in humans^5,13,31^. The expected two-year lag between a masting event and the increase in (infected) nymphs density has been confirmed by several studies^10,35–37^. Consistently, other studies highlighted such time lag between masting and the incidence of tick-borne disease including TBE and Lyme borreliosis^5,13,31,32^. In particular, some modelling efforts considering a beech masting index provided quite reliable predictions for the annual incidence of TBE in humans 2 years later^5,13,31^.

Climatic factors are not the only determinants of mast events^24,38^ and masting index is often obtained from a limited number of forest stands, or limited number of years of observation or dependent on the observer. For some forest tree species, such as beech and oak, airborne pollen amount is a key driver of seed production^39–41^, resulting in a link between pollen availability and masting. Since the presence and quantity of pollen in the air has a significant impact on allergies in humans, air quality is monitored at the global level to improve the prevention of allergic diseases. Hence, in this study we tested whether pollen abundance data provides an early warning predictor for TBE infection risk. To this aim, we investigated the correlation between pollen data, derived from a 30-year long term dataset, and TBE incidence in humans in an endemic area in northern Italy.

## Materials and methods

### Study area and pollen data

Pollen data were recorded at Fondazione Edmund Mach, in San Michele all’Adige, Province of Trento, Italy (Latitude 46.19 N, Longitude 11.13 E, 220 m a.s.l.), from 1989 to 2020. Airborne pollen was sampled by a Hirst-type sampler and analyzed following conventional techniques and standardized protocols (UNI EN 16868:2019). San Michele all’Adige is located near the center of the province of Trento, the area under study.

The study area is located in the alpine biogeographical region, and we considered the following forest tree species growing in the area: hop-hornbeam (*Ostrya carpinifolia* Scop.), beech (*Fagus sylvatica* L.), spruce (*Picea abies* L*.*), pine (*Pinus sylvestris* L. and *P. nigra* J. F. Arnold), downy oak (*Quercus pubescens* Willd.), manna ash (*Fraxinus ornus* L.) and hazel (*Corylus avellana* L.).

### TBE data

TBE is a notifiable disease in Europe since 2012 and in Italy since 2017. The case classification and definition are provided by the European Center for Disease Control^42^. The number of human clinical cases of TBE in the Province of Trento from 1992 to 2020 was provided by the local Public Health Agency (Azienda Provinciale per i Servizi Sanitari Provincia Autonoma di Trento, APSS). We denote the number of cases of TBE recorded during year *y* with ${N}_{TBE}(y)$. The yearly TBE incidence (number of cases per 100,000 population) ${I}_{TBE}(y)$ was calculated according to the number of inhabitants in the area during year y as per the national census data^43^.

### Statistical analysis

Total amount of pollen grains produced during the main pollen season (95% of the total^44^), denoted by *P*_*T*_(*y*), was calculated for each considered tree species *T* and each year *y*, applying a gap filling of missing data built on the basis of the seasonality of the historical database. Overall, only 4.18% of the data was interpolated.

The annual TBE incidence ${I}_{TBE}(y)$ was transformed prior to analysis in order to normalize its distribution following the Box-Cox method^45^. We denote by ${I}_{TBE}(y{)}^{*}$ the transformed variable, defined as

1 $$I_{TBE} \left( y \right)^{*} = \frac{{I_{TBE} \left( y \right)^{\lambda } - 1}}{\lambda } .$$

We standardized the pollen quantity collected during year* y* for each tree species *T* to standard scores by subtracting the mean and dividing by the standard deviation. This standardization ensures that the pollen quantity of each tree species is measured in standard deviations rather than in absolute amounts. The new quantity is thus defined as

2 $$P_{T} (y)^{*} = \frac{{P_{T} \left( y \right) - m\left( {P_{T} } \right)}}{{sd\left( {P_{T} } \right)}},$$

where m(*P*_*T*_) and sd(*P*_*T*_) represent the mean and the standard deviation of *P*_*T*_(*y*), respectively (values shown in the Supplementary Table S1 online).

We first investigated the association between ${I}_{TBE}(y{)}^{*}$ and the total amount of pollen of the tree species of interest collected during previous years (from *y−*1 to *y−*3 included) by developing univariate linear models which can be represented by the following equation:

3 $$I_{TBE} (y)^{*} = \beta_{0,T,y - n} + \beta_{T,y - n} \cdot P_{T} (y - n)^{*}$$

where β_0,T,y−n_ and β_T,y−n_ are the model coefficients (intercept and slope) and ${P}_{T}(y-n{)}^{*}$ represents the standardized total amount of pollen (see Eq. 2) of taxon *T* collected during year *y*−*n* (*n* = 1, 2, 3). Therefore, 21 models (7 taxa and 3 time lags) were evaluated. Afterwards, we built a full model by considering all covariates having a significant slope β_T,y-n_ in the univariate models previously described, after checking for potential collinearity between variables. In case of substantial correlation (Pearson correlation coefficient > 0.7^46^), we considered only the variable having the lowest Akaike Information Criterion (AIC) score in the associated univariate model.

All analysis was carried out using R v4.2.0^47^ and packages *AeRobiology*^48^, *EnvStats*^49^, *tidyverse*^50^ and *car*^51^. Transformed pollen quantities and incidences are presented in Supplementary Table S2 online.

## Results

**Figure 1:**
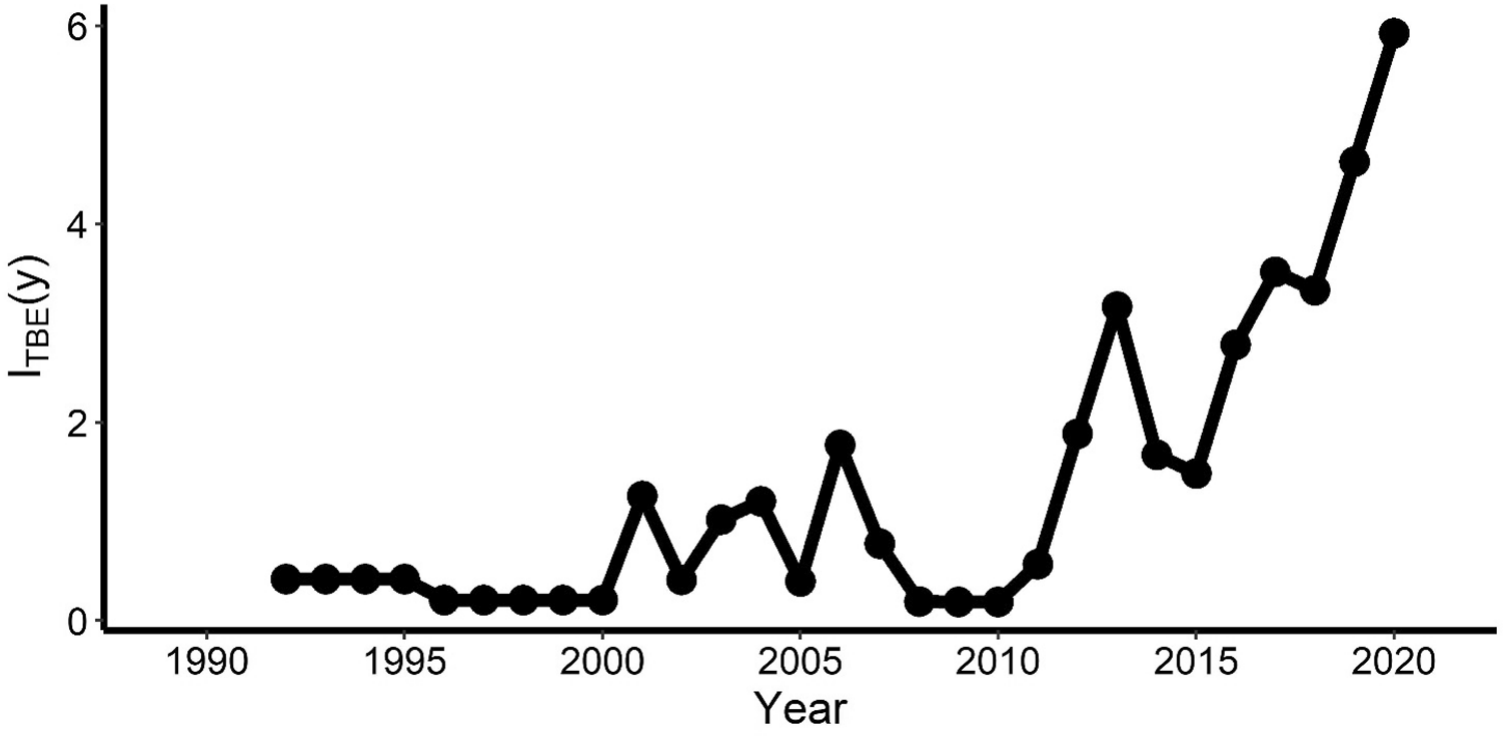
TBE incidence. Number of TBE human cases per 100,000 population per year (${I}_{TBE}(y)$) in the Province of Trento, Italy (1992–2020).

Between 1992 and 2020, a total of 206 TBE human infections were recorded in the province of Trento. The incidence varied between years, as shown in Fig. 1, with an apparent increasing trend from 2016 onwards despite the ongoing vaccination campaigns (vaccine has been offered freely to inhabitants of the region since 2018). The annual TBE incidence was transformed assuming λ = − 0.08 in Eq. (1).

**Figure 2:**
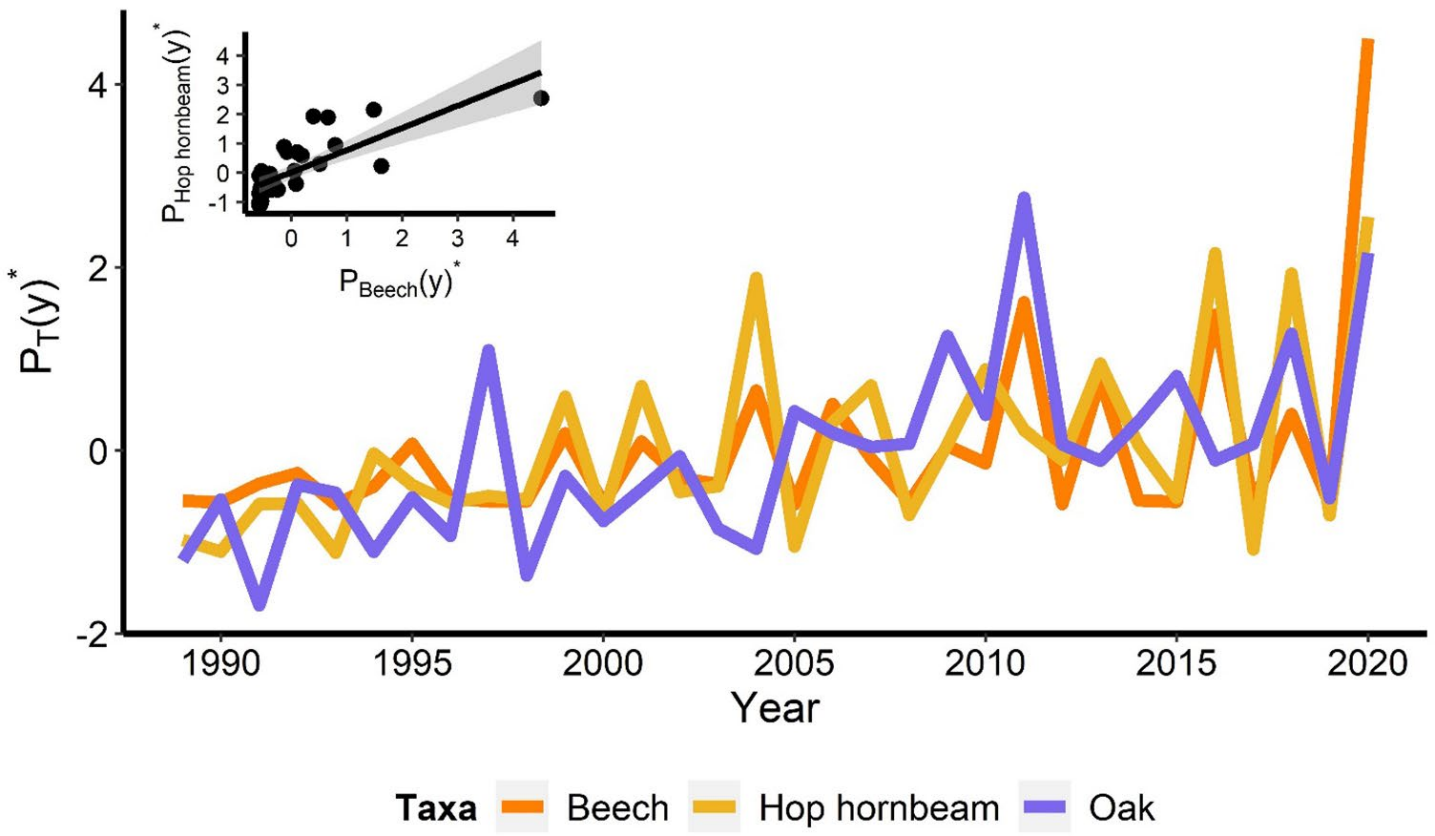
Pollen recorded quantities. Standardized total amount of pollen (${P}_{T}(y{)}^{*}$, see Eq. 2) for beech (*Fagus sylvatica* L.), oak (*Quercus pubescens* Willd.) and hop hornbeam (*Ostrya carpinifolia* Scop.). Inset: standardized quantities for beech (${P}_{Beech}(y{)}^{*}$, x-axis) and hop hornbeam (${P}_{Hop hornbeam}(y{)}^{*}$, y-axis).

We found a significant association between TBE incidence and pollen indicators with a two-year time lag for beech, oak and hop hornbeam (${P}_{T}(y{)}^{*}$ shown in Fig. 2). All other time lags and tree species resulted in non-significant relationships.

**Figure 3:**
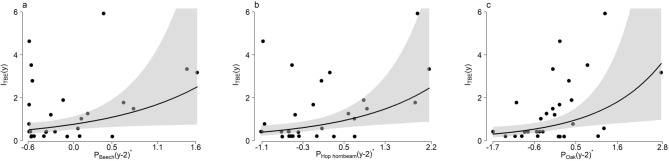
Univariate models. Relationship between the annual TBE incidence (number of cases per 100,000 population, y-axis) and the standardized total amount of recorded pollen per year with a 2-year lag (*y−*2) (x-axis) for beech (*Fagus sylvatica,* panel **a**), hop hornbeam (*Ostrya carpinifolia,* panel **b**) and oak (*Quercus* sp*.,* panel **c**). Lines: univariate model predictions (back-transformed) with confidence intervals (shaded areas). Dots: observed TBE incidence.

The coefficients of the selected univariate models (significant regression slopes) are presented in Table 1 while model predictions are shown in Fig. 3. Although all other investigated associations were not significant (*p* values > 0.05), we can note that they were almost always positive (see Supplementary Note online).

**Table 1:** Estimates, standard errors, t values and *p* values of the parameters of the univariate models (represented by Eq. 3) with significant intercepts (β_0,y−2_) and slopes (β_T,y−2_) and their R^2^ coefficients and AIC scores.

Taxon	Parameter	Coefficient estimate	Standard error	t value	*P* value
Beech (*Fagus sylvatica*; R^2^ = 0.15, AIC = 90.1)	β_0,y-2_	− 0.27	0.2	− 1.344	0.19
	β_T,y-2_	0.715	0.33	2.166	0.039
Hop hornbeam (*Ostrya carpinifolia*; R^2^ = 0.2, AIC = 88.3)	β_0,y-2_	− 0.341	0.192	− 1.77	0.088
	β_T,y-2_	0.559	0.215	2.593	0.015
Oak (*Quercus* sp.; R^2^ = 0.2, AIC = 88.4)	β_0,y-2_	− 0.304	0.193	− 1.573	0.128
	β_T,y-2_	0.551	0.214	2.573	0.02

Taking into account such findings, we built the full model considering ${P}_{T}(y-2{)}^{*}$ for hop hornbeam and oak pollen only, excluding beech, as ${P}_{Beech}(y{)}^{*}$ and ${P}_{Hop hornbeam}(y{)}^{*}$ were highly correlated (see Fig. 2; Pearson correlation coefficient = 0.77, *p* < 0.001). The model can thus be represented as

4 $$I_{TBE} (y)^{*} = \beta_{0} + \beta_{Oak,y - 2} \cdot P_{Oak} (y - 2)^{*} + \beta_{Hop\, hornbeam,y - 2} \cdot P_{Hop\, hornbeam} (y - 2)^{*}$$

This model (coefficients presented in Table 2) explained a higher fraction of the variation in the incidence of TBE compared to univariate models (R^2^ = 0.34) and, consistently with the previous analysis, both tree species are positively associated with TBE incidence.

**Table 2:** Estimates, standard errors, t values and *p* values of the parameters of the full model (see Eq. 4) for the transformed TBE incidence (${I}_{TBE}(y{)}^{*})$.

Parameter	Coefficient estimate	Standard error	t value	*P* value
β_0_ (intercept)	− 0.31	0.179	− 1.737	0.094
β_Hop hornbeam,y−2_	0.478	0.202	2.363	0.026
β_Oak,y−2_	0.47	0.2	2.342	0.027

We carried out a sensitivity analysis by excluding from the pollen dataset the total records for which more than 20% of the data were interpolated (eight ${P}_{T}(y{)}^{*}$ values, as reported in the Supplementary Table S1). Results did not change substantially, although the relationship between ${N}_{TBE}(y{)}^{*}$ and ${P}_{Beech}(y-2{)}^{*}$ became not significant (*p* = 0.057, full results presented in the Supplementary Note online).

## Discussion

In recent years, the incidence of tick-borne diseases in human populations has been rising rapidly^52,53^. Consequently, the availability of long-term, easily accessible, and standardized parameters that help to predict spatial and temporal occurrence of these tick-borne pathogens is becoming increasingly important. In this study, we investigated whether tree pollen quantities might be associated with the human incidence of TBE at different temporal lags. To the best of our knowledge, this is one of the first attempts at assessing this potential relationship.

The optimal ecological conditions for TBE viral circulation, and therefore the infection hazard, are not yet completely elucidated, but the abundance of vector ticks and reservoir animals are fundamental^2,20^. In this context, mast seeding has been widely considered to be an appropriate predictor of tick-borne diseases as it affects both the annual nymphal tick density^10,30,35,36^ and the inter-annual fluctuation in rodents populations^32,34,54^. In this regard, the 1-year lagged population density of small rodents (mainly *Apodemus* spp. and *Myodes* spp.) following a mast event has been repeatedly demonstrated to be correlated with simultaneous^33,55,56^ or postponed^12,30,35^ disease incidence fluctuations in humans. More specifically, it has been highlighted by several studies that tick-borne pathogens’ incidence is expected to be higher two years after a masting event^5,13,31,32^. For instance, in^5^ the authors used the beech masting index, among other predictors, to forecast human TBE incidence in Germany, Austria and Switzerland, but also underlined the importance of considering other forest tree species that are food resources for small mammals, such as oak (*Quercus* spp.) or European spruce (*Picea abies*). However, measuring seed production in long-lived plants requires direct observations over many years which may differ among countries adding intrinsic uncertainties to this value^57^. Moreover, such measures often consist of arbitrary categorical observations, which might not be easily compared as they are observer-dependent^58^.

Airborne pollen is measured around the world^59^ through national and supranational networks (e.g., http://www.pollnet.it/default_it.asp; https://www.uco.es/rea/; http://www.aaaai.org/global/nab-pollen-counts) with standardized databases mostly available and accessible for several tree taxa. The link between pollen amount and acorn production has received a lot of attention because fruiting dynamics dramatically impacts forest regeneration, biodiversity, population dynamics of seed consumers, and ultimately epidemiology of infectious disease. Many tree species, like oak and beech, which dominate temperate forests in Europe, regenerate through synchronized, highly variable fruit production^24,60–62^. Usually in these tree species, hot summer temperatures in the previous year affect flower initiation increasing pollination efficiency^63,64^, provided that the plants have enough energy (resource-limited floral induction model^65^). Regardless of this common feature, oak masting is also driven by pollen dynamics with mechanisms that involve both internal resource allocation and spring weather conditions that affect the amount of airborne pollen released (synchrony hypothesis^40,41^). Whatever the mechanism, the pulsed resource availability generated by inter-annual fluctuations in seed production eventually drives the population dynamics of granivore forest rodents^25^, by increasing the length of the rodent breeding season and enhancing their winter survival. Rodents are the main hosts of larval stages of ticks that will molt into nymphs the following year. This relationship is not always straightforward, although in the majority of the cases rodent density positively affects larval survival and abundance^9^. As a cascading effect, in forested areas given a certain prevalence of tick-borne pathogens in the host population, the density of infected nymphs and the risk of tick-borne disease transmission will increase.

In this study, TBE cases were strongly correlated with the pollen loads of oak, hop hornbeam and beech recorded two years earlier. The association with beech pollen was weaker (lower R^2^ and higher AIC), probably because of the distance of beech forests from the monitoring site, resulting in relatively small quantities of detected pollen, which might also be due to the intrinsic low dispersal capability of beech pollen, which consists of heavy and rapidly falling grains released simultaneously with the emergence of beech leaves^66^. The ratio of recorded pollen abundances among beech, oak and hop hornbeam were 1:9:61, respectively^67^. Pollen amounts of hop hornbeam and beech recorded at the sampling station during the study period showed highly synchronized fluctuations, and hop hornbeam was therefore a good proxy indicator for beech pollen and beech seed production.

Our findings are consistent with previous studies on the main predictors of tick-borne pathogens transmission in North America and Europe^5,13,31,32,35^ which highlighted a positive association between acorn abundance and number of cases recorded two years later. In particular, such relationship was observed in other Alpine areas (Austria and Switzerland) between TBE incidence and fructification 2 years prior^5,13,31^.

It should be noted that future predictions regarding mast seeding may be disrupted by anthropogenic environmental changes^68–70^, with potential effects on the community of seed consumers yet to be determined. A further limitation that prevents more accurate predictions is the lack of extensive data on TBE vaccination coverage among countries, including Italy.

Nevertheless, as pollen data collected using standardized procedures by widespread networks (over 800 monitoring stations in 2018^59^), the results of our small-scale, long-term study could be tested over larger areas. If the relationships are confirmed by further studies, airborne pollen data could be used as an early warning system for the risk of TBE in Europe where rodent dynamics are mostly driven by food availability. Public health agencies would know two years in advance of a potential TBE outbreak, which would give them time to plan preventive measures, such as campaigns aimed at raising public awareness and implementation of vaccination programs. Moreover, the proposed warning system could be tested and applied to other arthropod-borne infections, where pulsed resource availability could be estimated by airborne pollen and seed production.

## Supplementary Information

Supplementary Information 1.

Supplementary Information 2.

## Data Availability

All data generated or analyzed during this study are included in this published article and its supplementary information files.
