# Supplementary material for: Correlation between airborne pollen data and the risk of tick-borne encephalitis in northern Italy
Source: Sci Rep. 2023 May 22;13:8262. doi: 10.1038/s41598-023-35478-w (PMC10203324; doi:10.1038/s41598-023-35478-w)
Supplement: Supplementary file 1 — Supplementary Information 1. [file 41598_2023_35478_MOESM1_ESM.pdf]

Correlation between airborne pollen data and the risk of tick-borne encephalitis in northern Italy

Giovanni Marini[1], Valentina Tagliapietra[1], Fabiana Cristofolini[1], Antonella Cristofori[1], Francesca Dagostin[1], Maria Grazia Zuccali[2], Silvia Molinaro[2], Elena Gottardini[1], Annapaola Rizzoli[1]

[1]Research and Innovation Centre, Fondazione Edmund Mach, San Michele all'Adige (TN), Italy

[2] Azienda Provinciale Servizi Sanitari, Trento, Italy

Appendix

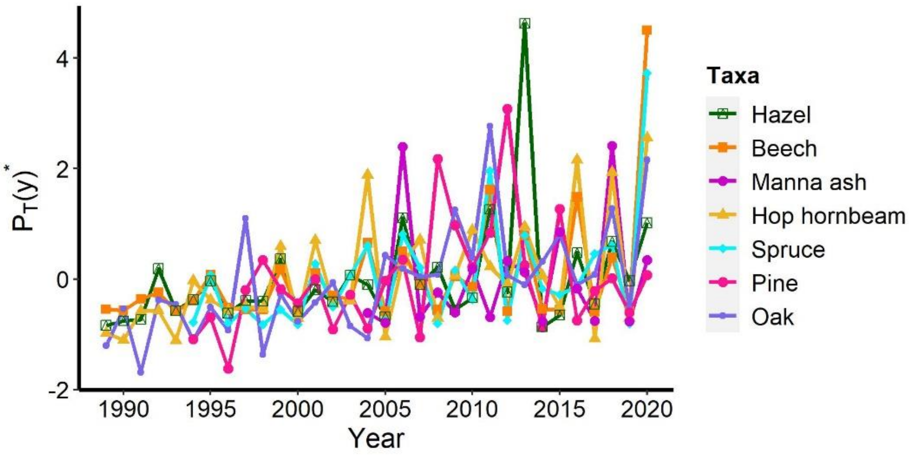

Figure A. Standardized total amount of pollen ($P_T(y)^*$, see equation (2) in the main text) recorded in San Michele all'Adige (Province of Trento, Italy) from 1989 to 2020 for each considered taxon (*T*).

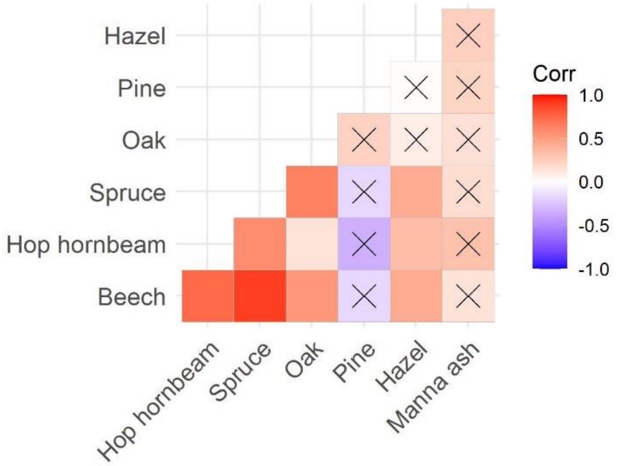

Figure B. Pearson correlation coefficients for each taxa pair of standardized total amount of pollen ($P_T(y)^*$). Crosses indicate non-significant associations (p-value>0.05).

Table A. Estimates, standard errors, t values and p-values of the parameters of the models associating $I_{TBE}(y)^*$ and $P_T(y-1)^*$.

Parameter	Coefficient Estimate	Standard Error	t value	P-value
Spruce (*Picea abies* L.)				
$\beta_{0,y-1}$	-0.238	0.236	-1.009	0.323
$\beta_{T,y-1}$	0.323	0.341	0.948	0.353
Hazel (*Corylus avellana* L.)				
$\beta_{0,y-1}$	-0.344	0.211	-1.634	0.114
$\beta_{T,y-1}$	0.225	0.208	1.08	0.29
Beech (*Fagus sylvatica* L.)				
$\beta_{0,y-1}$	-0.31	0.215	-1.441	0.161
$\beta_{T,y-1}$	0.304	0.353	0.862	0.396
Manna ash (*Fraxinus ornus* L.)				
$\beta_{0,y-1}$	0.156	0.301	0.518	0.612
$\beta_{T,y-1}$	0.227	0.296	0.766	0.456
Hop hornbeam (*Ostrya carpinifolia* Scop.)				
$\beta_{0,y-1}$	-0.342	0.213	-1.601	0.121
$\beta_{T,y-1}$	0.158	0.243	0.653	0.52
Pine (*Pinus sylvestris* L. and *P. nigra* J. F. Arnold)				
$\beta_{0,y-1}$	-0.272	0.235	-1.159	0.258
$\beta_{T,y-1}$	0.162	0.231	0.700	0.491
Oak (*Quercus pubescens* Willd.)				
$\beta_{0,y-1}$	-0.317	0.207	-1.532	0.137
$\beta_{T,y-1}$	0.347	0.229	1.514	0.142

Table B. Estimates, standard errors, t values and p-values of the parameters of the models associating $I_{TBE}(y)^*$ and $P_T(y-2)^*$.

**Parameter**	**Coefficient Estimate**	**Standard Error**	**t value**	**P-value**
Spruce (*Picea abies* L.)				
$\beta_{0,y-2}$	-0.194	0.229	-0.848	0.405
$\beta_{T,y-2}$	0.659	0.333	1.977	0.06
Hazel (*Corylus avellana* L.)				
$\beta_{0,y-2}$	-0.338	0.212	-1.598	0.122
$\beta_{T,y-2}$	0.187	0.207	0.902	0.375
Beech (*Fagus sylvatica* L.)				
$\beta_{0,y-2}$	-0.27	0.2	-1.344	0.19
$\beta_{T,y-2}$	0.715	0.33	2.166	0.039
Manna ash (*Fraxinus ornus* L.)				
$\beta_{0,y-2}$	0.234	0.32	0.731	0.478
$\beta_{T,y-2}$	-0.004	0.31	-0.012	0.991
Hop hornbeam (*Ostrya carpinifolia* Scop.)				
$\beta_{0,y-2}$	-0.341	0.192	-1.77	0.088
$\beta_{T,y-2}$	0.559	0.215	2.593	0.015
Pine (*Pinus sylvestris* L. and *P. nigra* J. F. Arnold)				
$\beta_{0,y-2}$	-0.251	0.243	-1.033	0.312
$\beta_{T,y-2}$	0.169	0.236	0.716	0.481
Oak (*Quercus pubescens* Willd.)				
$\beta_{0,y-2}$	β0,y-2	-0.304	0.193	-1.573
$\beta_{T,y-2}$	βT,y-2	0.551	0.214	2.573

Table C. Estimates, standard errors, t values and p-values of the parameters of the models associating $I_{TBE}(y)^*$ and $P_T(y-3)^*$.

**Parameter**	**Coefficient Estimate**	**Standard Error**	**t value**	**P-value**
Spruce (*Picea abies* L.)				
$\beta_{0,y\text{-}3}$	-0.141	0.244	-0.58	0.568
$\beta_{T,y\text{-}3}$	0.436	0.353	1.234	0.23
Hazel (*Corylus avellana* L.)				
$\beta_{0,y\text{-}3}$	-0.327	0.211	-1.550	0.133
$\beta_{T,y\text{-}3}$	0.213	0.206	1.036	0.31
Beech (*Fagus sylvatica* L.)				
$\beta_{0,y\text{-}3}$	-0.278	0.213	-1.307	0.202
$\beta_{T,y\text{-}3}$	0.473	0.347	1.362	0.184
Manna ash (*Fraxinus ornus* L.)				
$\beta_{0,y\text{-}3}$	0.182	0.339	0.537	0.601
$\beta_{T,y\text{-}3}$	-0.277	0.397	-0.696	0.5
Hop hornbeam (*Ostrya carpinifolia* Scop.)				
$\beta_{0,y\text{-}3}$	-0.297	0.205	-1.448	0.159
$\beta_{T,y\text{-}3}$	0.435	0.244	1.778	0.087
Pine (*Pinus sylvestris* L. and *P. nigra* J. F. Arnold)				
$\beta_{0,y\text{-}3}$	-0.194	0.237	-0.819	0.421
$\beta_{T,y\text{-}3}$	0.336	0.225	1.493	0.15
Oak (*Quercus pubescens* Willd.)				
$\beta_{0,y\text{-}3}$	-0.29	0.211	-1.378	0.179
$\beta_{T,y\text{-}3}$	0.326	0.234	1.391	0.176

Table D. Estimates, standard errors, t values and p-values of the parameters of the models associating $I_{TBE}(y)^*$ and $P_T(y-2)^*$ excluding pollen total records for which more than 20% of the data was interpolated.

**Parameter**	**Coefficient Estimate**	**Standard Error**	**t value**	**P-value**
Spruce (*Picea abies* L.)				
$\beta_{0,y-2}$	-0.154	0.233	-0.662	0.515
$\beta_{T,y-2}$	0.588	0.342	1.72	0.099
Hazel (*Corylus avellana* L.)				
$\beta_{0,y-2}$	1.277	0.216	5.916	<0.001
$\beta_{T,y-2}$	0.278	0.383	0.725	0.475
Beech (*Fagus sylvatica* L.)				
$\beta_{0,y-2}$	-0.235	0.203	-1.155	0.259
$\beta_{T,y-2}$	0.663	0.333	1.993	0.057
Hop hornbeam (*Ostrya carpinifolia* Scop.)				
$\beta_{0,y-2}$	-0.348	0.199	-1.745	0.093
$\beta_{T,y-2}$	0.55	0.224	2.456	0.021
Pine (*Pinus sylvestris* L. and *P. nigra* J. F. Arnold)				
$\beta_{0,y-2}$	1.476	0.226	6.523	<0.001
$\beta_{T,y-2}$	0.3	0.238	1.261	0.221
Oak (*Quercus pubescens* Willd.)				
$\beta_{0,y-2}$	-0.249	0.192	-1.297	0.206
$\beta_{T,y-2}$	0.56	0.209	2.679	0.013
